# Effects of a specific synbiotic blend on fecal short-chain fatty acids and gut inflammation in cow's milk-allergic children receiving amino acid–based formula during early life: results of a randomized controlled trial (PRESTO study)

**DOI:** 10.3389/falgy.2025.1667162

**Published:** 2025-11-27

**Authors:** Pantipa Chatchatee, Annelot C. Breedveld, Simone R. B. M. Eussen, Anna Nowak-Wegrzyn, Lars Lange, Suwat Benjaponpitak, Kok Wee Chong, Pasuree Sangsupawanich, Harm Wopereis, Manon M. Oude Nijhuis, Jane E. Langford, Atanaska I. Kostadinova, Valerie Trendelenburg, Robert Pesek, Carla M. Davis, Antonella Muraro, Michel Erlewyn-Lajeunesse, Adam T. Fox, Louise J. Michaelis, Kirsten Beyer

**Affiliations:** 1 Center of Excellence for Allergy and Clinical Immunology, Division of Allergy and Immunology, Department of Pediatrics, Faculty of Medicine, Chulalongkorn University, Bangkok, Thailand; 2 Danone Research & Innovation, Utrecht, Netherlands; 3 Allergy and Immunology, Department of Pediatrics, New York University Langone Health, New York, NY, United States; 4 St Marien Hospital, Bonn, Germany; 5 The Pediatric Allergy and Immunology Division, Department of Pediatrics, Faculty of Medicine Ramathibodi Hospital, Mahidol University, Bangkok, Thailand; 6 The Allergy Service, Department of Paediatric Medicine, KKWomen’s & Children’s Hospital, Singapore, Singapore; 7 The Department of Pediatrics, Faculty of Medicine, Prince of Songkla University, Hat Yai, Thailand; 8 The Department of Pediatric Pneumology, Immunology, and Intensive Care Medicine, Charité Universitätsmedizin Berlin, Berlin, Germany; 9 Arkansas Children’s Hospital, Little Rock, AR, United States; 10 Texas Children’s Hospital, Baylor College of Medicine, Houston, TX, United States; 11 The Food Allergy Referral Centre, Padua University Hospital, Padua, Italy; 12 University Hospital Southampton, Southampton, United Kingdom; 13 Guy’s and St Thomas’ NHS Foundation Trust, London, United Kingdom; 14 Great North Children’s Hospital, Newcastle Upon Tyne Hospitals NHS Foundation Trust, Newcastle Upon Tyne, United Kingdom

**Keywords:** cow's milk protein allergy, oral tolerance development, prebiotics, probiotics, synbiotics, amino acid-based formula, microbiota, short chain fatty acids

## Abstract

Consumption of an amino acid-based formula (AAF) with added synbiotics [short-chain oligofructose and long-chain inulin (scFOS/lcFOS, 9:1 ratio) and *Bifidobacterium breve* M-16V] (AAF-S) beneficially impacts the gut microbiome of infants with cow's milk allergy (CMA). We assessed the effect of consuming AAF with or without synbiotics by children with CMA for 12 months on their fecal (branched) short-chain fatty acids (SCFA/BCFA) concentrations, and on gut barrier and inflammation markers (Netherlands Trial Register NTR3725). Feces and saliva were collected from 161 children (≤13 months) with IgE-mediated CMA at baseline, 6 and 12 months after enrollment, and at 24 and 36 months follow-up. Fecal SCFA and BCFA were analyzed by gas chromatography, and gut barrier and inflammation markers were measured in saliva/feces by ELISA or ImmunoCAP. At 6 months, children receiving AAF-S had significantly lower fecal propionate, valerate and BCFA concentrations compared to children consuming AAF. The percentage of propionate from the total 6 SCFA/BCFA (acetate + butyrate + propionate + valerate + isobutyrate + isovalerate) was significantly lower, while the percentage of acetate from the total 6 SCFA/BCFA was significantly higher in the AAF-S group. There were no significant differences between groups in fecal concentrations of butyrate at 6 months, nor in SCFA or BCFA at baseline and after 12, 24 or 36 months. Intestinal inflammation and barrier markers did not differ between groups. Addition of synbiotics to AAF brings concentrations of key fecal microbial metabolites more in line with patterns observed in healthy breastfed infants. The effects on SCFA and BCFA concentrations were transient and only seen at 6 months.

## 1 Introduction

Exclusive breastfeeding is the optimal way to feed a baby for the first 6 months of life, providing health, social, economic, and environmental benefits. Breastmilk is the ideal source of nutrients, containing a large variety of components including human milk oligosaccharides (HMOs), microbiota, secretory IgA, lactoferrin, cytokines and hormones (1). A healthy gut microbiome – characterized in infancy by a high abundance of bifidobacteria along with a diverse and well-balanced array of microbes – significantly contributes to the health of infants and young children and helps to establish the basis for lifelong health (2, 3). Breastfeeding is the strongest determinant of the gut microbiome development in infancy (4, 5). Unfortunately, breastfeeding is not always possible resulting in the use of formulas. Formula-fed infants have an aberrant gut microbiome composition with a reduced number of bifidobacteria (6). Furthermore, other early life events, including cesarean section, antibiotic use, and pathogen exposure, can disturb the gut microbiome in early life. This so called microbiome dysbiosis is an imbalanced proportion of beneficial and pathogenic bacteria in which potentially pathogenic species are enriched and commensal species are lacking (7). Many studies [e.g., (8–17)] have shown that allergic children often present a dysbiotic gut microbiome including lower levels of bifidobacteria and lactobacilli. Mouse models using fecal microbiota transfer have shown that the fecal microbiota of infants with cow's milk allergy (CMA), in contrast to the fecal microbiota of healthy infants, exacerbates the allergic response in mice (18, 19). Disturbance of the gut microbiome homeostasis may affect immune development, and particularly immune cell regulation, resulting in impaired oral tolerance to dietary antigens (20). Studies have shown that diet, in particular dietary fibers and prebiotics, is able to modulate the composition and metabolic activity of the gut microbiome (21, 22), which in turn may contribute to improved intestinal barrier integrity and immune development (23).

To bring the microbiota composition of CMA infants closer to that of healthy breastfed infants, formula supplemented with specific human milk components can be considered. Prebiotic fructooligosaccharides (FOS) mimic the structure and function of human milk oligosaccharides present in breast milk (24). Furthermore, *Bifidobacterium breve* is the predominant bacterial species in breast milk and addition of *Bifidobacterium breve* to infant formula may contribute to bifidobacteria presence in the gut of formula-fed CMA infants (25).

We conducted a multi-center randomized controlled study (PRESTO) in which children aged ≤13 months with immunoglobulin E (IgE)-mediated CMA were randomly assigned to receive an amino acid-based formula (AAF) containing pre- and probiotics (synbiotics) or an identical AAF without synbiotics. We previously showed that addition of synbiotics to AAF (AAF-S) led to increased percentages of bifidobacteria and decreased percentages of the *Eubacterium rectale/Clostridium coccoides* group, i.e., a gut microbial composition closer to that seen in healthy, breastfed infants (26) (Supplementary Figure S1). Fewer children fed AAF-S were hospitalized due to infections as compared to those fed AAF without synbiotics. There was no effect of synbiotics on tolerance development; 49% of all children became tolerant to cow's milk at 12 months, with an additional 13% developing tolerance at 24 months, and 76% of all children tolerated cow's milk at 36 months (26).

In the present analyses, we focused on metabolites produced by the intestinal bacteria after fermentation of prebiotic fibers, i.e., short-chain fatty acids (SCFA). They play a critical role in establishing and maintaining intestinal health, mainly serving as important fuel for intestinal epithelial cells thereby supporting gut motility and intestinal barrier integrity (27). Yet, SCFAs exert systemic benefits beyond those seen in the gut, influencing glucose homeostasis, appetite regulation, energy expenditure and immunomodulation, for example through directly effecting T regulatory cells (28, 29). The major SCFA that arise in the colon after fermentation of dietary fibers are acetate (C2), propionate (C3) and butyrate (C4), together making up 90%–95% of the total SCFA present in the colon (30). Valerate (C5) levels are noticeably lower (31).

Branched SCFA (BCFA), predominantly isobutyrate and isovalerate, are generated through fermentation of branched chain amino acids (valine, leucine and isoleucine) after undigested protein reaches the colon (31). They are suggested as markers of colonic protein fermentation (32) and have been linked to increased production of ammonia and other metabolites that may cause intestinal cell damage (31, 33). Yet, the relevance of low BCFA concentrations on gastrointestinal health is poorly understood (34, 35). Diet affects fecal BCFA levels; a high protein diet results in higher BCFA concentrations, whereas high intakes of complex carbohydrates, including dietary fibers, lead to lower BCFA concentrations (33, 36–38).

In this study, we investigated fecal SCFA and BCFA in children with IgE-mediated CMA according to the consumption of AAF with or without synbiotics for 12 months. Additionally, we measured fecal pH, lactic acid levels and explored intestinal inflammation and barrier integrity markers in feces and saliva.

## 2 Methods

### 2.1 Study design and participants

The PRESTO Study is a multicenter, prospective, double-blind, randomized controlled trial (Netherlands Trial Register NTR3725) that investigated cow's milk tolerance development after 12 months consumption of an AAF containing synbiotics (26). The synbiotic blend consisted of prebiotic FOS and a probiotic strain. The FOS was a mixture of chicory-derived neutral oligofructose and long-chain inulin (scFOS/lcFOS) (BENEO-Orafti SA, Oreye, Belgium; 9:1 ratio) at a total concentration of 0.63 g/100 ml formula, and the probiotic strain was a *Bifidobacterium breve* M-16V (Morinaga Milk Industry, Tokyo, Japan) at a concentration of 1.47 × 10^9^ colony forming units/100 ml formula. The prebiotic FOS mixture mimics key functional aspects of HMOs, which are known to promote a bifidobacteria-dominated gut microbiota in breastfed infants (39). The 9:1 ratio reflects the short- to long-chain oligosaccharide ratio in HMOs, supporting both rapid and sustained fermentation in the colon (40). *Bifidobacterium breve* is the predominant bacteria in human milk and addition of this strain in combination with prebiotic FOS to infant formula may promote bifidobacteria abundance in the gut of cow's milk allergic formula-fed infants (25, 26). The synbiotic blend has been proven safe and efficacious, and shown to partially restore a breastfed-like microbiota profile in CMA infants and to influence metabolic activity and immune markers (11, 41–44).

The study was conducted in accordance with the World Medical Association Declaration of Helsinki and the International Conference on Harmonization guidelines for Good Clinical Practice, and approved by the relevant Institutional Ethics Committees. Full details of the study design are reported elsewhere (26). In summary, participants were ≤13 months old children with IgE-mediated CMA, confirmed by an open or double-blind placebo-controlled food challenge for cow's milk (CM), or anaphylaxis after CM ingestion and confirmation by 2 independent physicians (26). Eligible subjects were randomly allocated to receive an AAF containing the synbiotic blend (AAF-S) or a similar AAF without synbiotics (AAF). Both formulas were produced by Danone (Liverpool, United Kingdom). Caregivers were instructed to provide subjects with a minimum daily study product intake, i.e., 450 ml for 0- to 8-month-olds, 350 ml for 9- to 18-month-olds and 250 ml for >18-month-olds, along with an age-appropriate milk-free diet. The intervention period lasted 12 months with assessments scheduled at baseline and 6, 12 (end of intervention period), 24 and 36 (follow-up period) months after the start of the intervention.

### 2.2 Lab procedures for fecal and saliva markers

Feces and saliva were collected at baseline, and 6, 12, 24 and 36 months after the start of the study at least 60 min after the last feeding. Fresh feces was collected in a stool collection tube and saliva was sampled using a SalivaBio Children's Swab Device (Salimetrics, LLC., Carlsbad, USA). Fecal and saliva samples were immediately frozen and kept at −80 °C during transport to, and storage at the laboratory (Danone Research & Innovation, Utrecht, The Netherlands).

After thawing, fecal samples were diluted 1:10 in ice-cold Phosphate-buffered saline (PBS) and homogenized by vigorous shaking for 5 min (Multi ReaxTM) in presence of glass beads. Samples were centrifugated for 3 min at 15.000 g at 4 °C and clear supernatant was collected.

After thawing, swabs were centrifugated, and saliva was collected in an EDTP tube containing Protease inhibitor cocktail (Complete Ultra tablets mini; Roche, Basel, Switzerland). After mixing by vortex, the saliva was centrifugated for 10 min at 10.000 g at 4 °C. Collected supernatants were used for the different analyses described below or stored at −80 °C for future analyses.

Acetate, butyrate, propionate, valerate, isovalerate and isobutyrate concentrations were analyzed in supernatants of fecal homogenates by gas chromatography as previously described (45). Fecal pH was measured using a pH meter and lactic acid was measured with an enzymatic assay as previously described (46). Fecal markers of intestinal inflammation and barrier integrity, including eosinophil derived neurotoxin (EDN), calprotectin, Alpha1-antitrypsin (A1AT), and eosinophil cationic protein (ECP), were studied. EDN [EDN ELISA Kit (Immundiagnostik AG, Bensheim, Germany)], calprotectin [Bühlmann Calprotectin ELISA kit (Bühlmann Laboratories AG, Schönenbuch, Switzerland)] and A1AT [Human A1AT ELISA kit (Immunology Consultants Laboratory, Inc., Portland, USA)] were determined by Enzyme Linked Immunosorbent Assays (ELISA) following manufacturer's instructions except for sample dilutions (800×, 50–100× and 10.000× respectively for EDN, calprotectin and A1AT). ECP concentration in the fecal samples was measured with ImmunoCAP on a Phadia250 (Thermo Fisher Scientific, Uppsala, Sweden) according to protocol (dilution 10×). Secretory immunoglobulin A (sIgA), a marker for intestinal barrier integrity and upper gastrointestinal barrier function was measured in supernatants of fecal homogenates and saliva, respectively following an in-house validated ELISA (dilution 10.000–100.000x). The methods have been described in more detail before (47, 48).

### 2.3 Statistical analyses

Statistical analyses were performed on the all-subjects randomized data set, which covered all participants in the all-subjects treated data set defined as all subjects who received at least one sip of the study product. Descriptive statistics of all outcome parameters are reported as median (Q1–Q3), separately for the AAF-S and AAF study groups. For statistical comparisons between the study groups (AAF-S vs. AAF) in SCFA, BCFA, intestinal inflammation and barrier markers, and sIgA in saliva, Wilcoxon rank sum test was performed. For subgroup analyses no statistical analyses were performed due to low group numbers. *P*-values <0.05 were considered statistically significant. Statistical analyses were performed using SAS Enterprise Guide v4.3 or higher software for Windows (SAS Institute, Cary, NC). Graphs were prepared using Graphpad Prism 9.5.0 for Windows (GraphPad Software, San Diego, California, USA, https://www.graphpad.com).

## 3 Results

### 3.1 Subject characteristics and flow

A total of 169 subjects with confirmed IgE-mediated CMA were randomized. Baseline demographics, clinical characteristics and medical history are listed in Supplementary Table S1. Feces and saliva were collected from 161 subjects, of which 76 received AAF-S and 85 received AAF. Mean ± SD age of the subjects at baseline was 9.35 ± 2.36 months and 72% were male. Supplementary Figure S2 summarizes the flow of the subjects from baseline to 36 months after the start of the intervention.

### 3.2 pH, lactic acid and (branched) short-chain fatty acids

**Figure 1:**
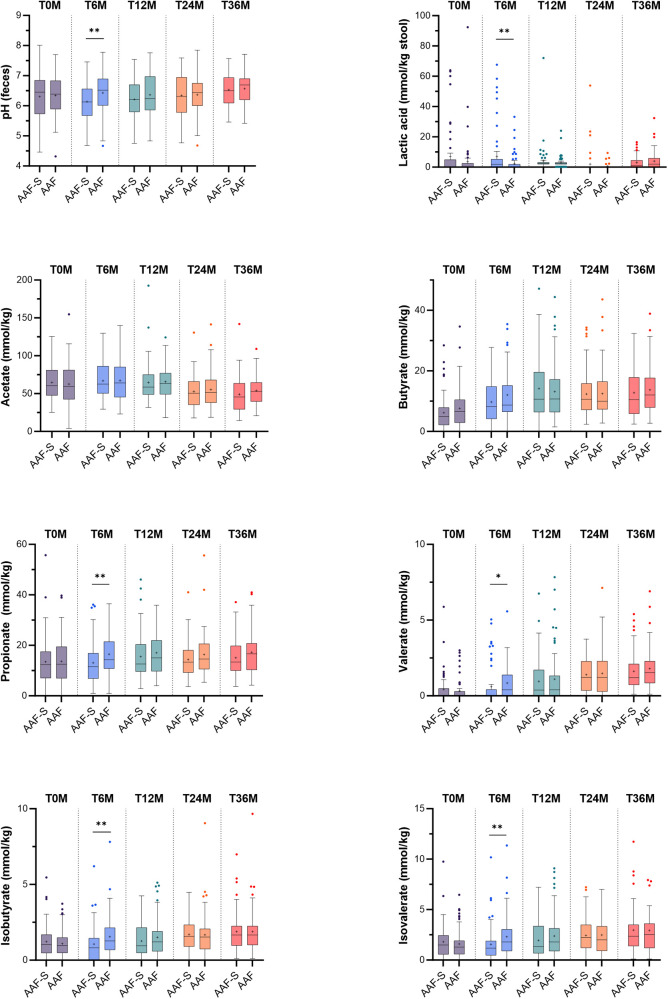
Boxplots with median, mean (+), quantiles (Q1–Q3), minimum and maximum, and outliers (•) of fecal pH, lactic acid and fecal short chain fatty acids or fecal branched chain fatty acids (in mmol/kg) at baseline, and 6, 12, 24 and 36 months after study initiation in children who received amino acid-based formula with synbiotics (AAF-S) or amino acid-based formula without synbiotics (AAF) for 12 months. * *p* ≤ 0.05, ** *p* ≤ 0.01.

**Figure 2:**
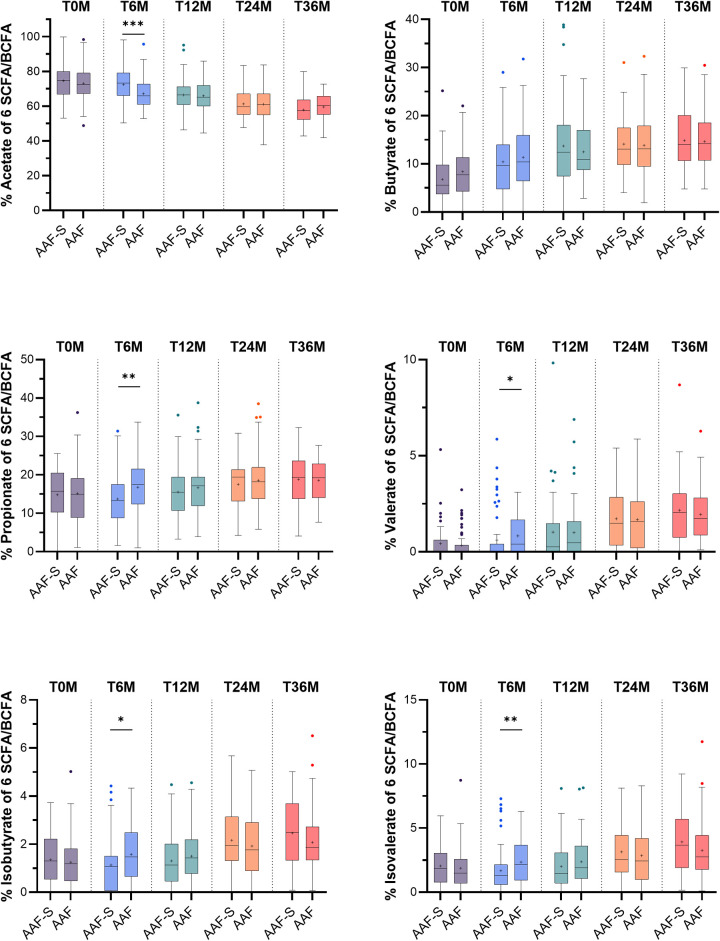
Boxplots with median, mean (+), quantiles (Q1–Q3), minimum and maximum, and outliers (•) of fecal short chain fatty acids or fecal branched chain fatty acids as percentage of 6 SCFA/BCFA (acetate + butyrate + propionate + valerate + isobutyrate + isovalerate) at baseline, and 6, 12, 24 and 36 months after study initiation in children who received amino acid-based formula with synbiotics (AAF-S) or amino acid-based formula without synbiotics (AAF) for 12 months. * *p* ≤ 0.05, ** ≤ 0.01, *** *p* ≤ 0.001.

Stool pH [median (IQR)] was significantly lower in the AAF-S group compared to the AAF group at 6 months [6.1 (5.7–6.6) vs. 6.5 (6.0–6.9), *p* = 0.004], but not at later time points (Figure 1, Supplementary Table S2). Lactic acid levels in stool were significantly increased in the AAF-S compared to the AAF group at 6 months only [1.9 mmol/kg (0.2–5.2) vs. 0.2 mmol/kg (0.2–1.9), *p* = 0.002] (Figure 1). The median percentage of acetate from the total of 6 SCFA/BCFA (acetate + butyrate + propionate + valerate + isobutyrate + isovalerate) was significantly higher in the AAF-S group at 6 months compared to the AAF group [73.2% (66.1–79.1) vs. 65.9% (61.2–72.6), *p* < 0.001] (Figure 2). Propionate, valerate, isobutyrate and isovalerate concentration and percentage of propionate, valerate, isobutyrate and isovalerate from the total of 6 SCFA/BCFA were significantly lower at 6 months in children receiving the AAF-S compared to those receiving AAF (propionate: 11.5 mmol/kg (6.8–16.8) vs. 14.3 mmol/kg (10.8–21.3), *p* = 0.004; 13.4% (8.8–17.5) vs. 17.5% (12.6–21.5), *p* = 0.002, valerate: 0.0 mmol/kg (0.0–0.4) vs. 0.4 mmol/kg (0.0–1.4), *p* = 0.016; 0.0% (0.0–0.4) vs. 0.4% (0.0–1.7), *p* = 0.045, isobutyrate: 0.8 mmol/kg (0.0–1.4) vs. 1.3 mmol/kg (0.7–2.1), *p* = 0.010; 1.1% (0.1–1.5) vs. 1.5% (0.7–2.5), *p* = 0.016, isovalerate: 1.2 mmol/kg (0.5–1.9) vs. 1.8 mmol/kg (0.9–3.0), *p* = 0.004); 1.3% (0.6–2.1) vs. 2.1% (0.9–3.6), *p* = 0.005) (Figures 1, 2). There were no significant differences between groups in fecal concentrations of acetate and butyrate, and proportion of butyrate at 6 months, nor in SCFA or BCFA at baseline and after 12, 24 or 36 months (Figures 1, 2, Supplementary Table S2). Total SCFA/BCFA concentrations did not differ between the AAF-S and AAF groups (Supplementary Figure S3). Subgroup analyses showed that infants in the AAF-S group enrolled ≤6 months of age had a higher percentage of acetate and a lower percentage of butyrate and isovalerate from the total of 6 SCFA/BCFA, as well as a lower concentration of butyrate and isovalerate at 6 months as compared to infants enrolled >6 months of age (Supplementary Figure S4).

### 3.3 Intestinal inflammation and barrier markers, and salivary sIgA

**Figure 3:**
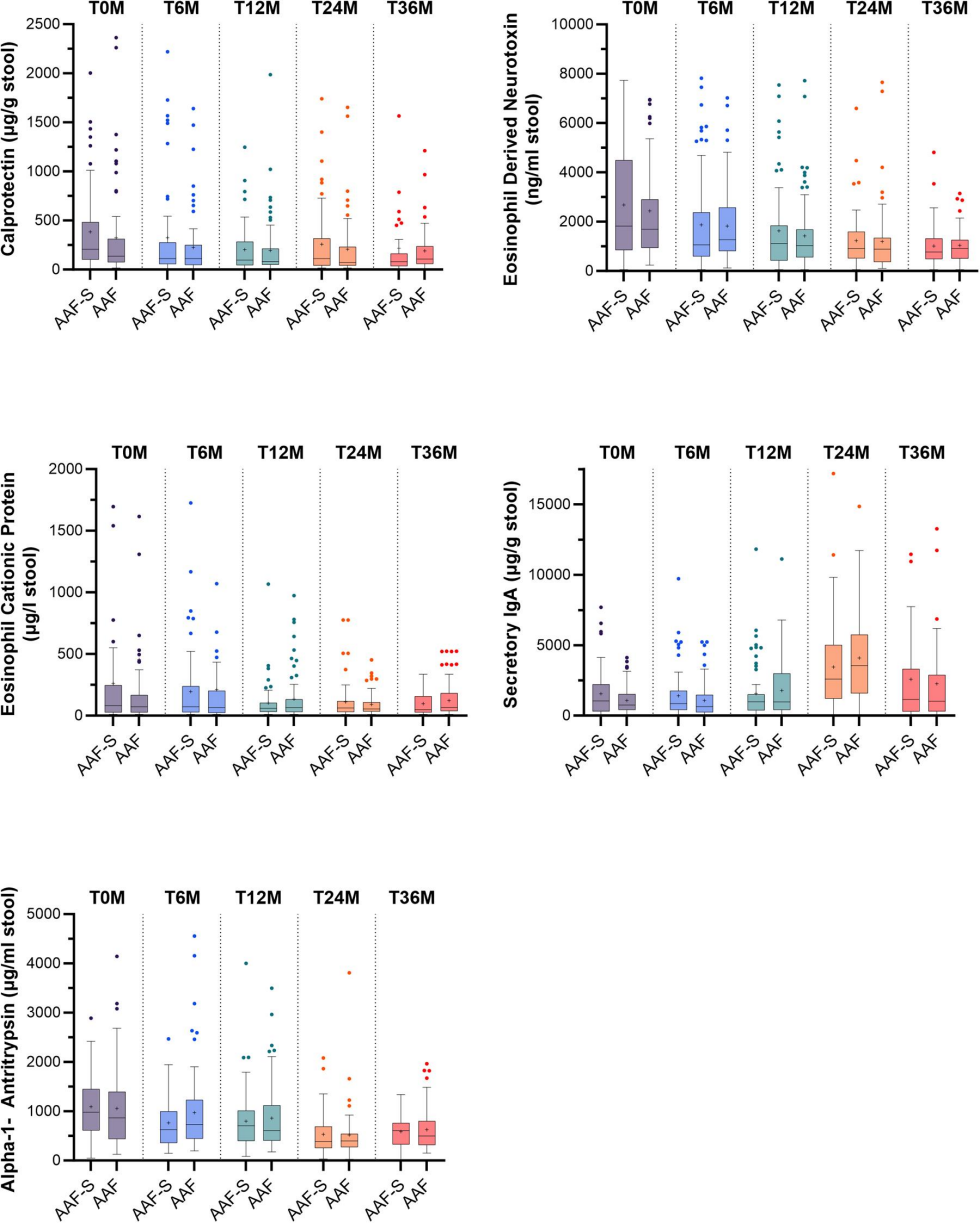
Boxplots with median, mean (+), quantiles (Q1–Q3), minimum and maximum, and outliers (•) of intestinal inflammation and barrier markers at baseline, and 6, 12, 24 and 36 months after study initiation in children who received amino acid-based formula with synbiotics (AAF-S) or amino acid-based formula without synbiotics (AAF) for 12 months. ** *p* ≤ 0.01.

**Figure 4:**
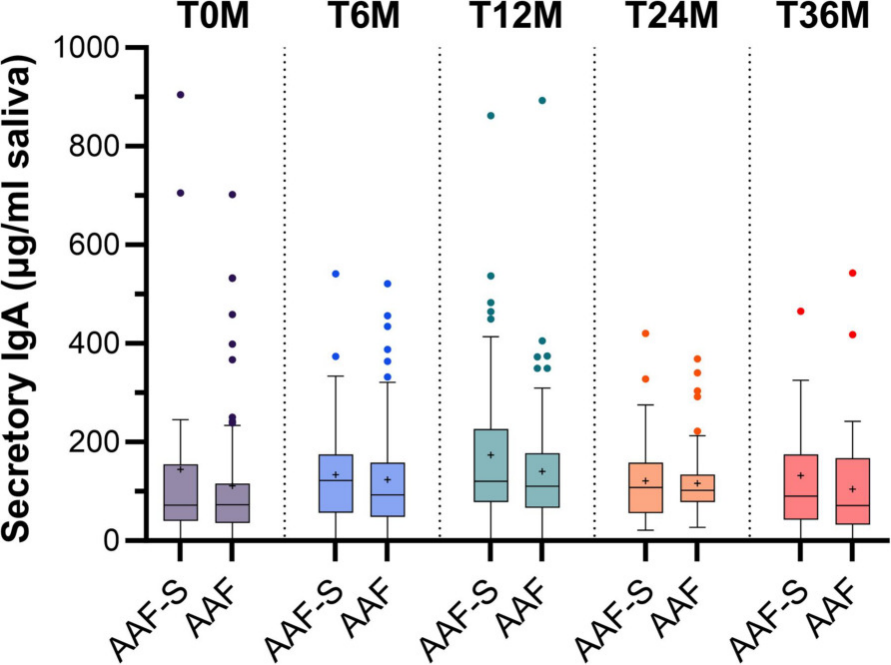
Boxplots with median, mean (+), quantiles (Q1–Q3), minimum and maximum, and outliers (•) of secretory IgA in saliva at baseline, and 6, 12, 24 and 36 months after study initiation in children who received amino acid-based formula with synbiotics (AAF-S) or amino acid-based formula without synbiotics (AAF) for 12 months.

There were no significant differences between the AAF-S and AAF groups in intestinal inflammation and barrier integrity markers at any of the timepoints (Figure 3), nor in salivary secretory IgA levels (Figure 4). Neither subgroup analyses by age (≤6 vs. >6 months at inclusion) demonstrated pronounced differences (Supplementary Figure S5).

## 4 Discussion

Addition of synbiotics to AAF has been shown to improve the gut microbiota of children with IgE-mediated CMA (11, 42) (Supplementary Figure S2). This study evaluated the impact of synbiotic consumption as part of AAF over a 12-month intervention period on fecal SCFA and BCFA concentrations, and intestinal inflammation and barrier integrity markers. Although there was no significant difference in acetate concentration between the AAF-S and AAF groups, the percentage of acetate from the total of 6 SCFA/BCFA was significantly higher in the AAF-S group at 6 months. Expressing individual SCFA/BCFA as a percentage of the total pool captures shifts in the relative distribution of fermentation products. This compositional perspective is important, because changes in SCFA/BCFA ratios can influence host physiology, gut pH, and microbial ecology, even when total levels remain stable (49, 50). Subgroup analyses demonstrated lower concentrations and percentage of butyrate and isovalerate, but higher percentage of acetate in infants enrolled at ≤6 months. This suggests that younger infants (≤6 months) differently metabolize synbiotics compared to older infants (>6 months) resulting in a distinct metabolite profile.

Acetate is the most abundant SCFA in the colon and has been described to have anti-inflammatory and anti-infective effects (51, 52) and to enhance IgA production and IgA's reactivity to the microbiota (53–55). Our data suggest the presence of a positive association in the change from baseline at 6 months in the proportion of acetate with the concentration of fecal sIgA. This association seems stronger in children receiving AAF-S (*r* = 0.3818, *p* = 0.0022) as compared to children receiving AAF (*r* = 0.1257, *p* = 0.2893) (Supplementary Figure S6).

Concentrations and proportions of fecal propionate were significantly lower in children receiving AAF-S for 6 months compared to those receiving AAF. Likewise, concentrations of valerate, isobutyrate and isovalerate, were significantly lower in children receiving the AAF-S. A higher proportion of acetate and lower concentrations of propionate, valerate, isobutyrate and isovalerate, have been reported in exclusively breastfed infants compared to those receiving no or partial breastfeeding (56–58). Yet, although the addition of synbiotics to AAF brought concentrations of the SCFA/BCFA closer to those found in healthy breastfed infants, still differences are noticeable. Bridgman et al. reported higher acetate and lower butyrate, propionate, isobutyrate and isovalerate concentrations in healthy exclusively breastfed infants compared to the concentrations in children receiving AAF-S in our study. The SCFA/BCFA profile observed in breastfed infants can be attributed, at least partly, to the presence of HMOs in breast milk. HMOs reach the colon intact where they serve as selective growth substrates for bifidobacteria (52, 59). The prebiotic mix of scFOS/lcFOS (ratio 9:1), as present in the AAF-S, mimics the function, structure, and concentration of the HMOs in human milk. However, HMOs have additional complexity and diversity which, together with other human milk components (including lactoferrin and IgA), can account for the different SCFA/BCFA levels observed in healthy exclusively breastfed infants in the Bridgman study. Furthermore, the fecal SCFA profile in healthy breastfed infants changes with increasing age, being characterized by elevated acetate levels in the first 6 months of life. Propionate and butyrate increase after 8–10 months of age when solid foods are introduced, and breastfeeding is less or discontinued (60). Infants included in the study of Bridgman et al. were ∼4 months old, whereas the initial measurements of SCFA/BCFA concentrations in the present study were conducted at ∼15 months of age. Consequently, this comparison should be interpreted with caution, as differences in SCFA/BCFA profiles cannot be exclusively attributed to breastfeeding; age-related changes and feeding practices are also known to influence these metabolite levels.

The significant differences in SCFA/BCFA levels between the AAF-S and AAF study groups were only observed 6 months after the start of the intervention, and were not present after 12 months (end of intervention period), nor after 24 or 36 months (follow-up period). These results are in line with a previous study in younger healthy infants (9–16 weeks old) who received cow's milk based infant formula supplemented with short-chain galacto-oligosaccharides (scGOS)/lcFOS and B. breve M-16V for 6 weeks. SCFA levels were closer to those observed in healthy breastfed infants, but this effect was partly abolished after study product intake was discontinued (61).

The similar SCFA/BCFA profile at the end of the intervention is probably explained by the shift of the gut microbiome towards a more adult-like gut microbiome characterized by higher percentages of bacteria from the *Firmicutes* and *Bacteroidetes* phyla as the child grows older, has lower formula intake, and eats a more adult-type diet (62).

The significantly elevated lactic acid levels and higher percentage of acetate in children receiving AAF-S can be attributed to the elevated proportion of bifidobacteria as previous reported for this study (26) (Supplementary Figure S1). Lactic acid produced by bifidobacteria leads to a reduction in pH which may inhibit the growth of opportunistic pathogens such as *Clostridiaceae*, *Enterobacteriaceae* and *Staphylococcaceae* (52, 59, 63). The lower fecal pH of children receiving AAF-S is in line with observations in healthy breastfed infants (57) and previous studies where dietary synbiotics were studied (61, 64–67). These results support the idea that synbiotics not only bring the gut microbiota composition of formula-fed CMA infants closer to that observed in breastfed infants, but also more closely aligns its metabolic activity and intestinal milieu.

We also measured concentrations of fecal intestinal inflammation and barrier integrity markers and salivary secretory IgA. Calprotectin, an inflammatory marker derived from neutrophils, has immunomodulatory and antimicrobial properties. Fecal calprotectin levels correlated positively with cow's milk-related symptom score (68), and CMA infants on a strict cow's milk protein (CMP)-free diet had levels within the healthy range (69, 70). No differences in fecal calprotectin levels were found between children fed AAF-S or AAF, and levels remained within the range for healthy children (71).

Alpha1-antitrypsin, resistant to intestinal proteolysis, can extravasate from the serum to the gut lumen when intestinal permeability increases. This selective permeability helps train the immune system, potentially reducing allergy risks later in life (72). Alpha1-antitrypsin levels were significantly higher in breastfed infants than in formula-fed ones, possibly due to its presence in human milk (73). In our study, fecal alpha1-antitrypsin levels varied between individuals and were unaffected by synbiotic supplementation, being similar to previously reported levels (74).

EDN and ECP, markers of eosinophil activation, have cytotoxic and neurotoxic properties. CMA infants had (non-significant) higher fecal EDN levels before starting an elimination diet compared to healthy infants, with high variability (75). Fecal EDN levels varied between subjects and were not different between the AAF-S and AAF groups, however, a decreasing trend over time was observed. Those decreasing EDN levels align with increased CMP tolerance, with 49%, 62% and 76% of all children developing tolerance at 12, 24 and 36 months, respectively (26). This decrease may be due to age and CMP tolerance development. Fecal ECP levels did not differ between the AAF-S and AAF group, which is consistent with a previous study in which children with non-IgE mediated CMA (<13 months old) received an AAF with or without synbiotics for 26 weeks (42). Significantly higher levels of ECP were found in 6-month-old infants who were exclusively breastfed compared to those exclusively formula-fed (76). This may be the result of ECP present in human milk (77).

Secretory IgA plays a crucial role in neutralizing pathogens and regulating the microbiota composition (78). No differences were found in fecal and salivary secretory IgA levels between the AAF-S and AAF groups. Increased fecal and salivary sIgA levels were found in breastfed infants compared to formula-fed infants (78–81). Fecal and salivary sIgA levels measured in the CMA children included in our study were comparable to those found in healthy formula-fed children (82, 83). Supplementing infant formula with prebiotic GOS and/or FOS led to increased fecal sIgA levels in healthy infants included directly after birth (47, 83) or at 0–4 months of age (84), whereas probiotic supplementation resulted in highly variable fecal sIgA levels similar to those in infants receiving formula without probiotics (83, 85). The lack of early-life intervention in our study may explain why increased fecal sIgA levels were not observed as participants missed key immunomodulatory properties of pre- and probiotics. Furthermore, human milk contains sIgA and other immune-modulating components which drive immune maturation and potentially account for differences in sIgA levels between formula- and breastfed infants (86).

The transient nature of the synbiotic effect may be attributed to several factors. First, cessation of the intervention at 12 months likely contributed to the attenuation of benefits as the microbiota reverted towards baseline composition. Second, complementary feeding may have influenced the outcome of this study. The estimated energy- and protein-intake from complementary feed did not differ between the AAF and AAF-S group (data not shown), suggesting that the complementary feed (composition) is not a confounder. However, a reduction in formula intake when the children grow older may explain that effects seen at the 6-month time point gradually disappear over time. Study product adherence was similar between the AAF and AAF-S groups with >85% adherence at 2 weeks, increasing to >91% at 3, 6, 9 and 12 months after study start (data not shown). The median age of the children at inclusion was ∼9 months, meaning that the first post-intervention measurement occurred at ∼15 months of age. The composition of complementary feed can influence fecal SCFA/BCFA composition, potentially diluting the impact of the synbiotic blend. Adult omnivores tend to have increased propionate, valerate, isovalerate, and isobutyrate levels, whereas vegans tend to have elevated butyrate and acetate levels (87, 88). Third, the maturation of the infant gut microbiota may reduce responsiveness to the intervention over time.

The relatively high baseline age is a limitation of this study and can be attributed to the fact that IgE-mediated CMA is usually diagnosed between 4 and 6 months of age. Studies assessing microbiota and metabolites would benefit from including infants diagnosed with CMA very early in life (e.g., before 3 months of age) and to follow-up regularly after the intervention has started, to capture the synbiotic effect on early-life microbiota and immune programming and limiting bias from complementary feeding. Future studies may also benefit from documenting the age at which complementary feeding begins.

The production of SCFA/BCFA was measured in fecal samples. This only represents levels in the final part of the digestive system, not necessarily in other sections of the colon. Furthermore, these levels do not directly indicate production by the gut microbiota, as absorption processes are also involved. For future studies, it might be useful to measure SCFA/BCFA levels in serum, as well as amounts that have been absorbed and become systemically available.

Mixed model analyses showed similar outcomes where data fit the model. However, due to inadequate fit for some parameters, mixed model results are not presented. Overall, the results of this study should be interpreted with caution given its exploratory nature and the absence of multiplicity adjustments.

## 5 Conclusion

Early intervention in CMA children with synbiotics provides an opportunity to reprogram the intestinal microbiome. Synbiotic supplementation may be used as adjunct therapy in CMA to support a healthy intestinal habitat. Furthermore, prolonged intervention with synbiotics may provide sustained benefits on intestinal health and beyond. Implementing a multi-omics approach would give a holistic view of the biological systems and may help unravel the mechanism of action of dietary interventions. This remains to be explored in future studies.

## Data Availability

The original contributions presented in the study are included in the article/Supplementary Material, further inquiries can be directed to the corresponding author.

## Supplementary material

Supplementary Figure 1 Boxplots with median, mean (+), quantiles (Q1–Q3), minimum and maximum, and outliers (•) of percentages of bifidobacteria (left) and *Eubacterium rectale* and *Clostridium coccoides* (ER/CC) (right) at baseline and at 6, 12, 24 and 36 months after study initiation in children who received amino acid-based formula with synbiotics (AAF-S) or amino acid-based formula without synbiotics (AAF) for 12 months. ** *p* ≤ 0.01, *** p ≤ 0.001.

Supplementary Figure 2 Consort diagram showing the flow of subjects in the AAF-S and AAF study arms. AAF: amino acid-based formula, AAF-S: amino acid-based formula with added synbiotics (prebiotic mixture of short-chain oligofructose and long-chain inulin in 9:1 ratio and probiotic strain *Bifidobacterium breve* M-16V), M: months.

Supplementary Figure 3 Boxplots with median, mean (+), quantiles (Q1–Q3), minimum and maximum, and outliers (•) of acetate + butyrate + propionate + valerate + isobutyrate + isovalerate (=total SCFA/BCFA) concentration (in mmol/kg) in stool at baseline, and 6, 12, 24 and 36 months after study initiation in children who received amino acid-based formula with synbiotics (AAF-S) or amino acid-based formula without synbiotics (AAF) for 12 months.

Supplementary Figure 4 Boxplots with median, mean (+), quantiles (Q1–Q3), minimum and maximum, and outliers (•) of fecal short chain fatty acids or fecal branched chain fatty acids (in mmol/kg) (left) and fecal short chain fatty acids or fecal branched chain fatty acids as percentage of 6 SCFA/BCFA (acetate + butyrate + propionate + valerate + isobutyrate + isovalerate) (right) at baseline, and 6, 12, 24 and 36 months after study initiation in children who received amino acid-based formula with synbiotics (AAF-S) or amino acid-based formula without synbiotics (AAF) for 12 months subdivided in children enrolled ≤6 or >6 months of age. Post hoc subgroup analysis: statistical analyses were not conducted due to small group numbers (AAF-S *n* = 7, AAF *n* = 10) in ≤6 months of age groups.

Supplementary Figure 5 Boxplots with median, mean (+), quantiles (Q1–Q3), minimum and maximum, and outliers (•) of pH, lactic acid, intestinal inflammation and barrier markers and secretory IgA in saliva at baseline, and 6, 12, 24 and 36 months after study initiation in children who received amino acid-based formula with synbiotics (AAF-S) or amino acid-based formula without synbiotics (AAF) for 12 months subdivided in children enrolled ≤6 or >6 months of age. Post hoc subgroup analysis: statistical analyses were not conducted due to small group numbers (AAF-S *n* = 7, AAF *n* = 10) in ≤6 months of age groups.

Supplementary Figure 6 Correlation between the percentage of acetate from the total of 6 short chain fatty acids/branched chain fatty acids (acetate + butyrate + propionate + valerate + isobutyrate + isovalerate) and fecal secretory IgA (ug/g stool) at 6 months after study initiation in children who received amino acid-based formula with synbiotics (AAF-S, red line) or amino acid-based formula without synbiotics (AAF, blue line) for 12 months. Baseline values were subtracted. Post hoc analysis: statistics Pearson r correlation: AAF-S group; *p* = 0.0022, *r* = 0.3818), AAF group; *p* = 0.2893, *r* = 0.1257.
