# Supplementary figures and images for: Effects of a specific synbiotic blend on fecal short-chain fatty acids and gut inflammation in cow's milk-allergic children receiving amino acid–based formula during early life: results of a randomized controlled trial (PRESTO study)
Source: Front Allergy. 2025 Nov 27;6:1667162. doi: 10.3389/falgy.2025.1667162 (PMC12695744; doi:10.3389/falgy.2025.1667162)

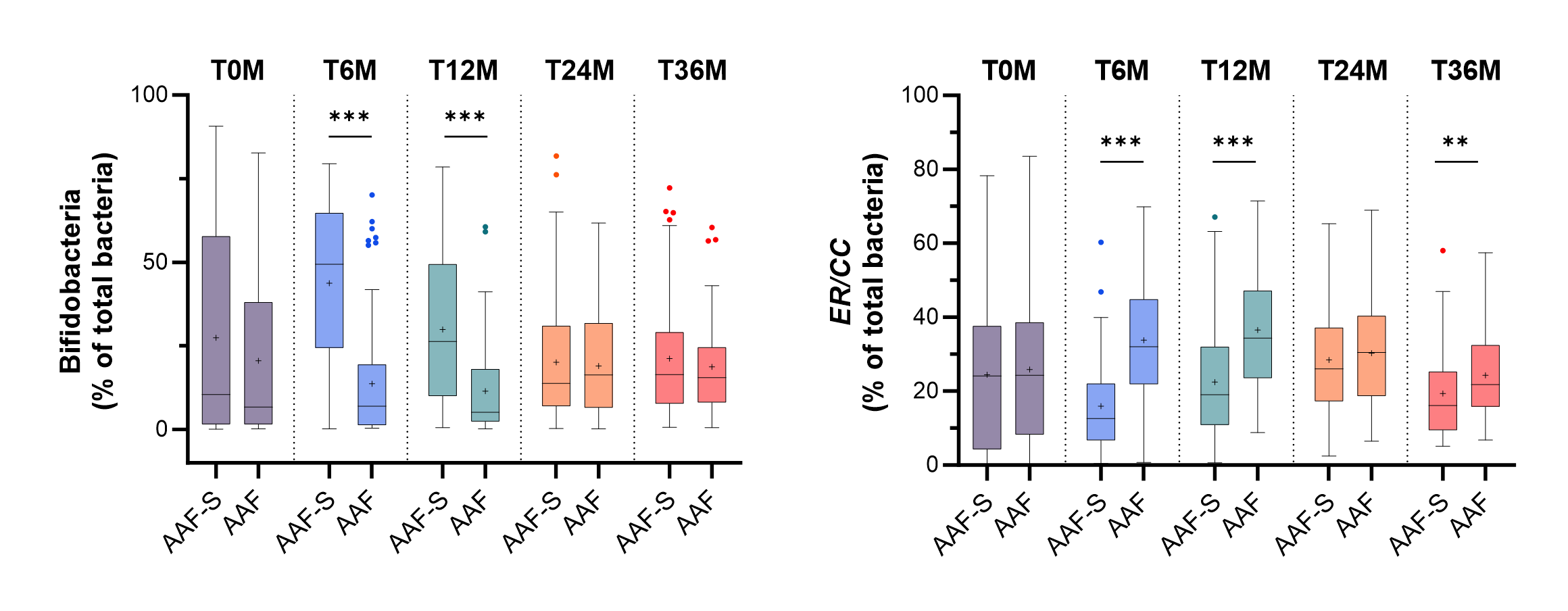

Supplement: Supplementary Figure 1 — Boxplots with median, mean (+), quantiles (Q1–Q3), minimum and maximum, and outliers (•) of percentages of bifidobacteria (left) and Eubacterium rectale and Clostridium coccoides (ER/CC) (right) at baseline and at 6, 12, 24 and 36 months after study initiation in children who received amino acid-based formula with synbiotics (AAF-S) or amino acid-based formula without synbiotics (AAF) for 12 months. ** p ≤ 0.01, *** p ≤ 0.001. [file Image1.tif]

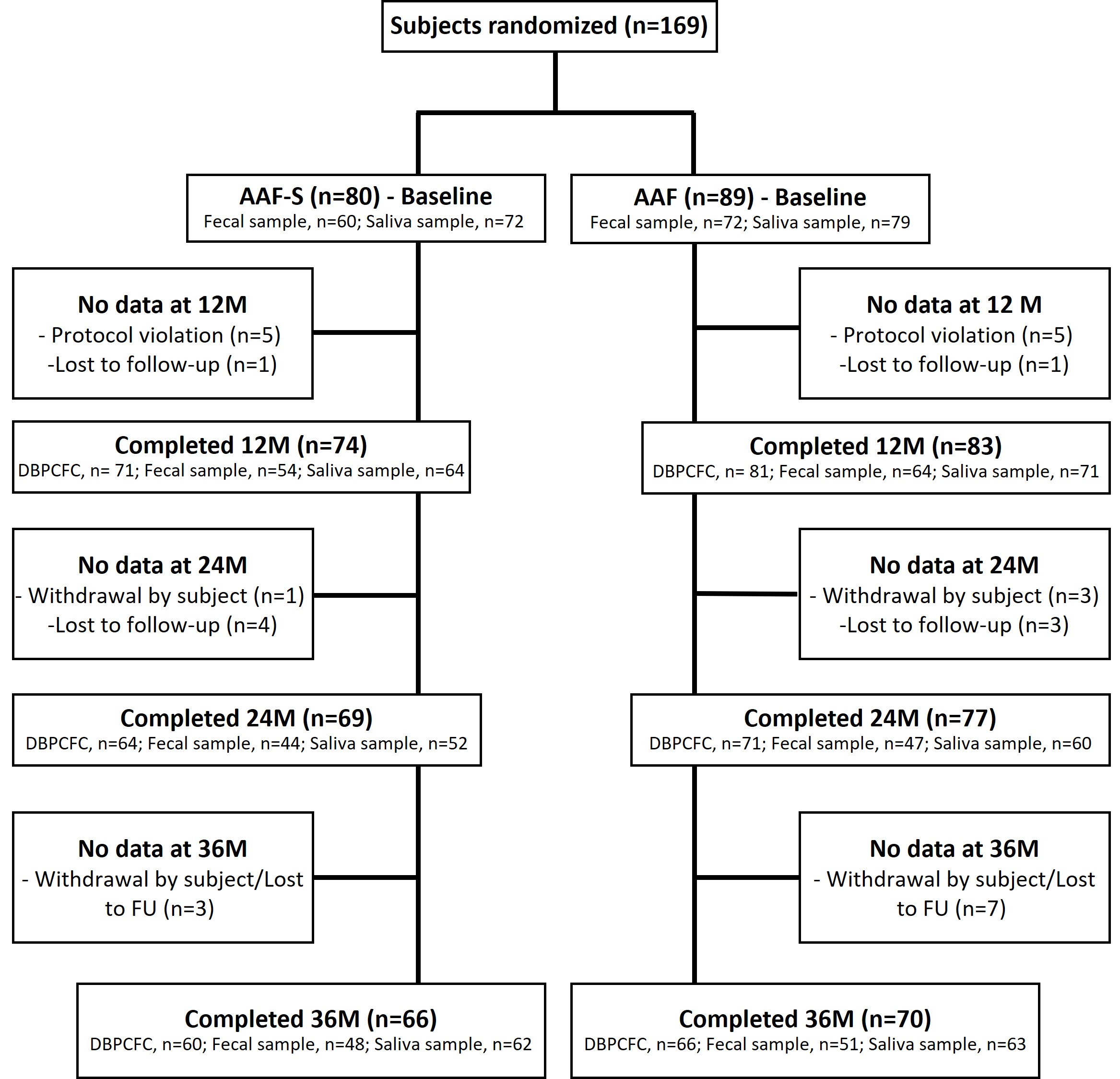

Supplement: Supplementary Figure 2 — Consort diagram showing the flow of subjects in the AAF-S and AAF study arms. AAF: amino acid-based formula, AAF-S: amino acid-based formula with added synbiotics (prebiotic mixture of short-chain oligofructose and long-chain inulin in 9:1 ratio and probiotic strain Bifidobacterium breve M-16V), M: months. [file Image2.jpeg]

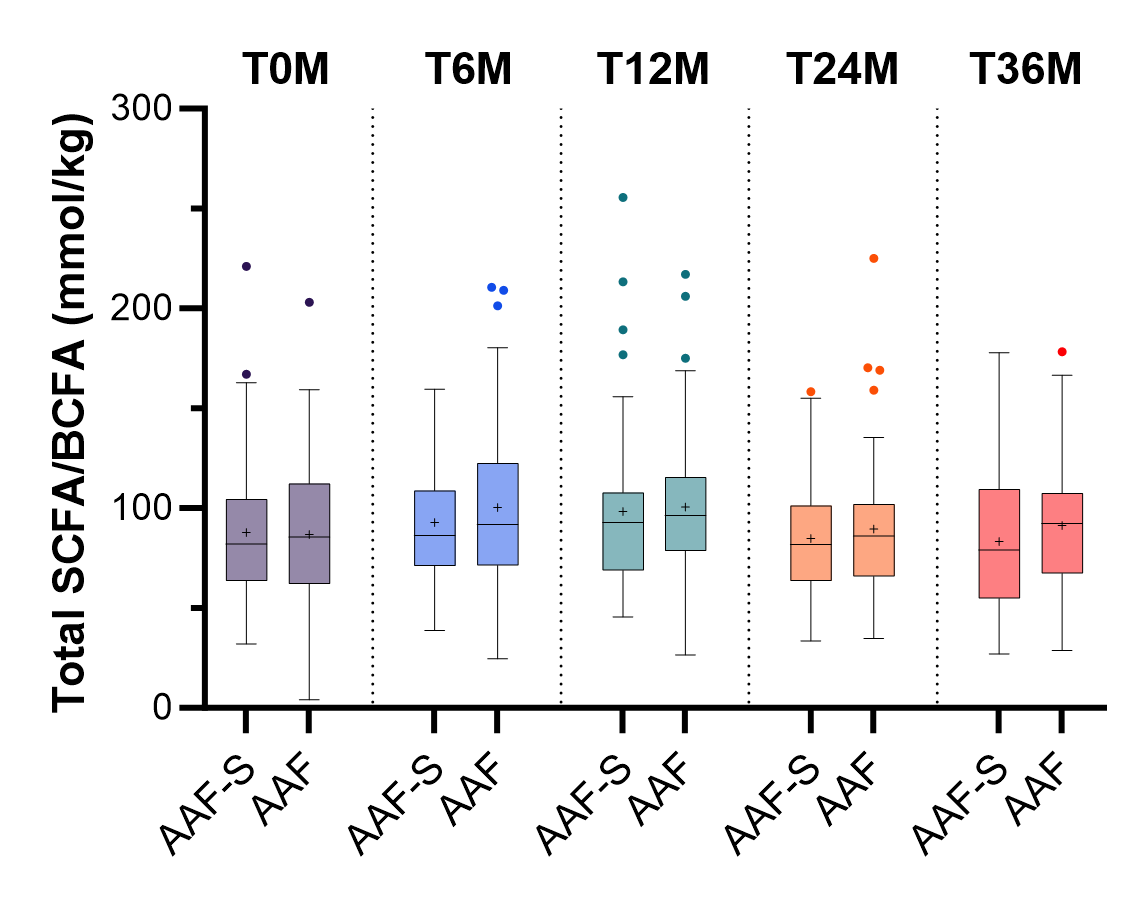

Supplement: Supplementary Figure 3 — Boxplots with median, mean (+), quantiles (Q1–Q3), minimum and maximum, and outliers (•) of acetate + butyrate + propionate + valerate + isobutyrate + isovalerate (=total SCFA/BCFA) concentration (in mmol/kg) in stool at baseline, and 6, 12, 24 and 36 months after study initiation in children who received amino acid-based formula with synbiotics (AAF-S) or amino acid-based formula without synbiotics (AAF) for 12 months. [file Image3.tif]

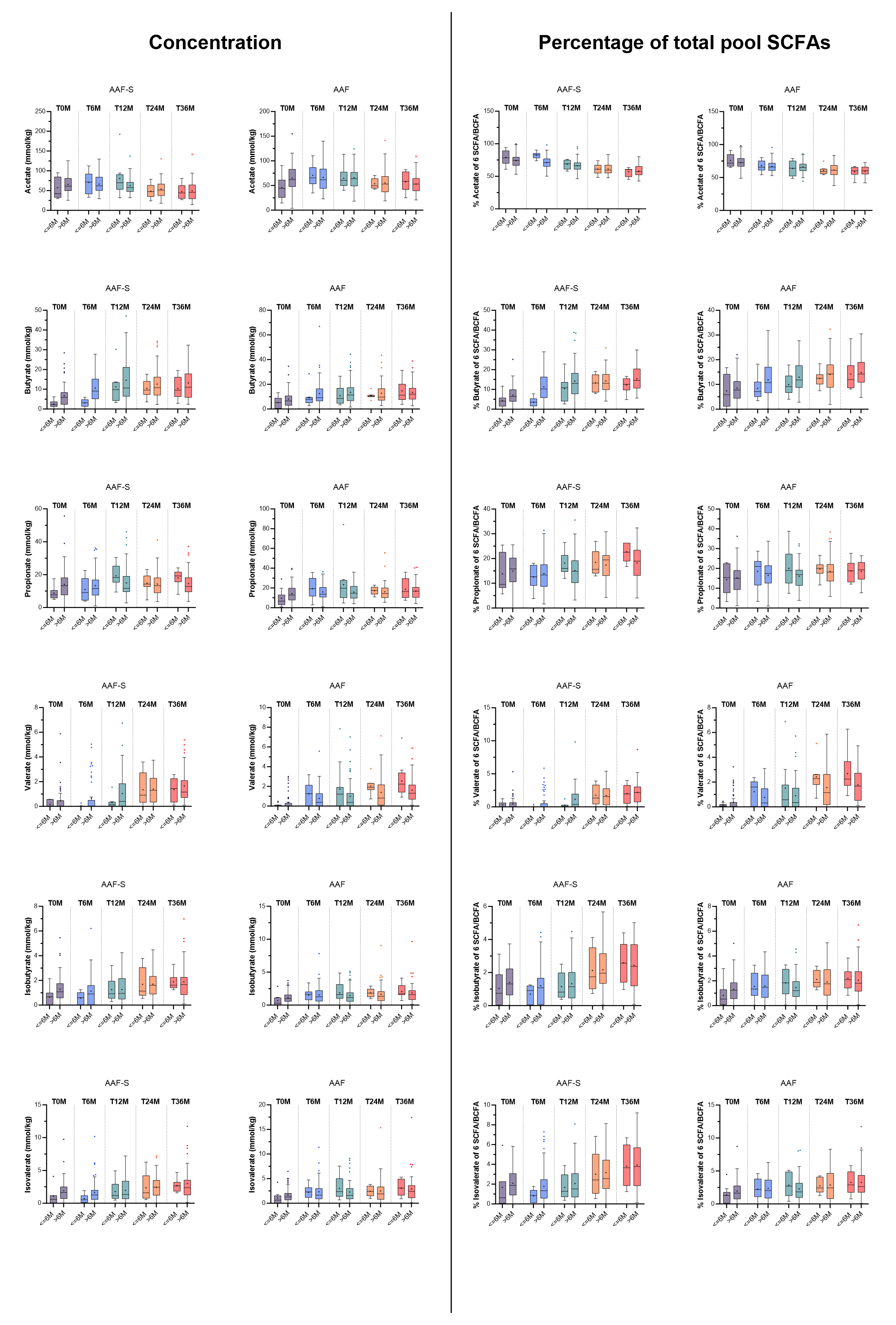

Supplement: Supplementary Figure 4 — Boxplots with median, mean (+), quantiles (Q1–Q3), minimum and maximum, and outliers (•) of fecal short chain fatty acids or fecal branched chain fatty acids (in mmol/kg) (left) and fecal short chain fatty acids or fecal branched chain fatty acids as percentage of 6 SCFA/BCFA (acetate + butyrate + propionate + valerate + isobutyrate + isovalerate) (right) at baseline, and 6, 12, 24 and 36 months after study initiation in children who received amino acid-based formula with synbiotics (AAF-S) or amino acid-based formula without synbiotics (AAF) for 12 months subdivided in children enrolled ≤6 or >6 months of age. Post hoc subgroup analysis: statistical analyses were not conducted due to small group numbers (AAF-S n = 7, AAF n = 10) in ≤6 months of age groups. [file Image4.tif]

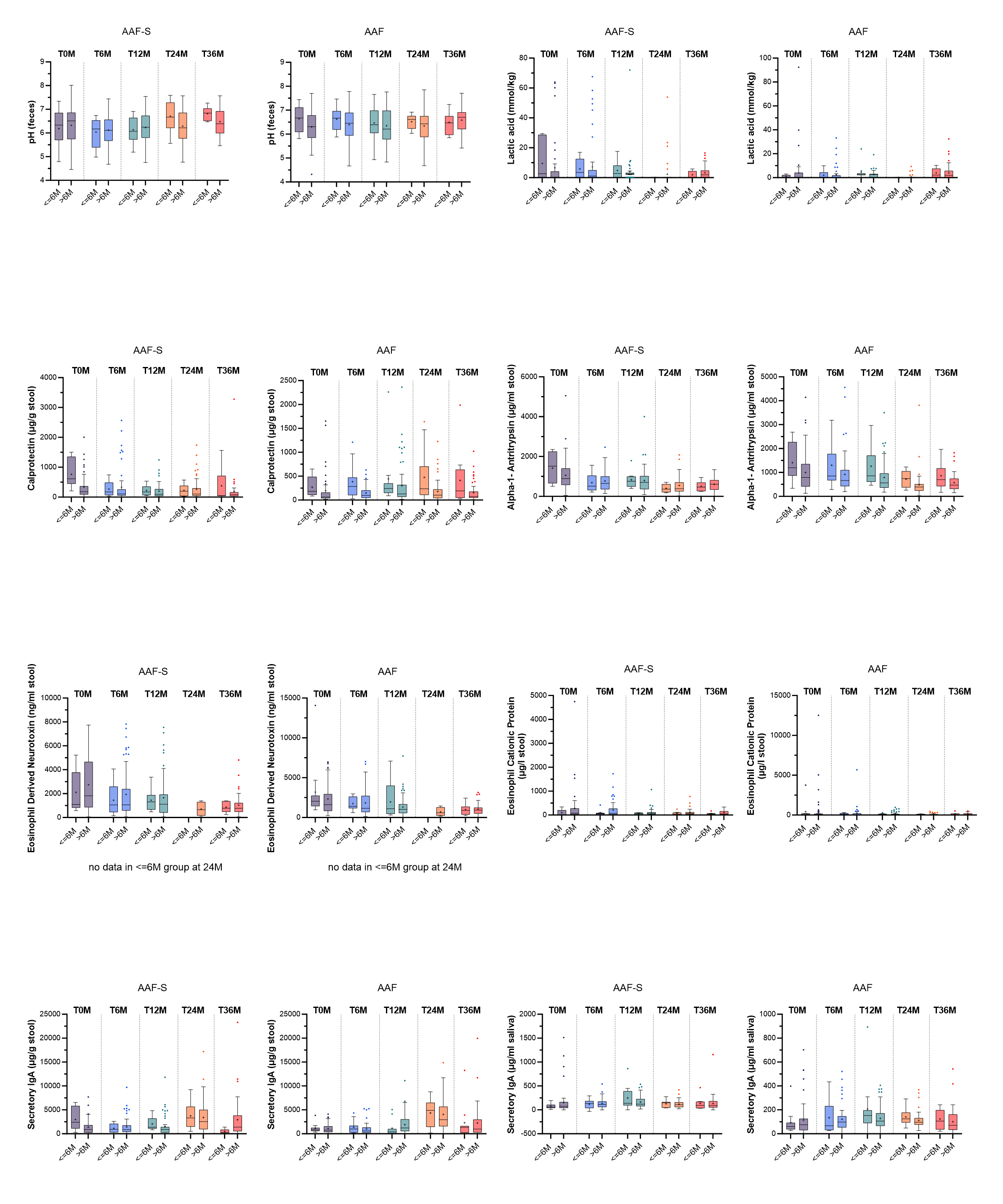

Supplement: Supplementary Figure 5 — Boxplots with median, mean (+), quantiles (Q1–Q3), minimum and maximum, and outliers (•) of pH, lactic acid, intestinal inflammation and barrier markers and secretory IgA in saliva at baseline, and 6, 12, 24 and 36 months after study initiation in children who received amino acid-based formula with synbiotics (AAF-S) or amino acid-based formula without synbiotics (AAF) for 12 months subdivided in children enrolled ≤6 or >6 months of age. Post hoc subgroup analysis: statistical analyses were not conducted due to small group numbers (AAF-S n = 7, AAF n = 10) in ≤6 months of age groups. [file Image5.tif]

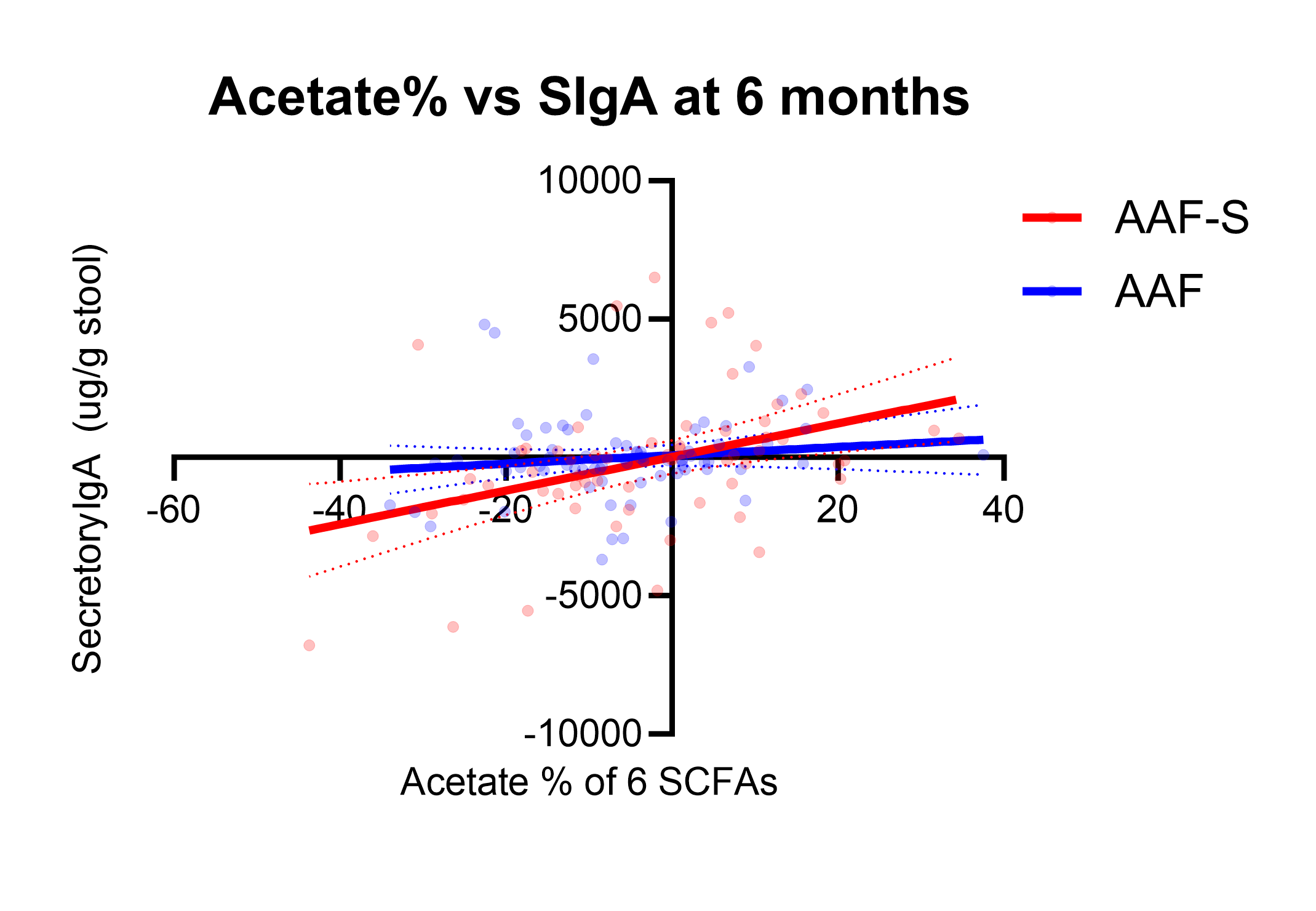

Supplement: Supplementary Figure 6 — Correlation between the percentage of acetate from the total of 6 short chain fatty acids/branched chain fatty acids (acetate + butyrate + propionate + valerate + isobutyrate + isovalerate) and fecal secretory IgA (ug/g stool) at 6 months after study initiation in children who received amino acid-based formula with synbiotics (AAF-S, red line) or amino acid-based formula without synbiotics (AAF, blue line) for 12 months. Baseline values were subtracted. Post hoc analysis: statistics Pearson r correlation: AAF-S group; p = 0.0022, r = 0.3818), AAF group; p = 0.2893, r = 0.1257. [file Image6.tif]
