# Supplementary material for: Effects of a specific synbiotic blend on fecal short-chain fatty acids and gut inflammation in cow's milk-allergic children receiving amino acid–based formula during early life: results of a randomized controlled trial (PRESTO study)
Source: Front Allergy. 2025 Nov 27;6:1667162. doi: 10.3389/falgy.2025.1667162 (PMC12695744; doi:10.3389/falgy.2025.1667162)
Supplement: Supplementary file 7 [file Datasheet1.pdf]

Supplementary Table 1. Baseline characteristics.

Characteristic	AAF-S (n = 80)	AAF (n = 89)	Total (n = 169)
Age at baseline (months), mean ± SD	9.39 ± 2.29	9.33 ± 2.74	9.36 ± 2.53
Sex			
Male	57 (71)	65 (73)	122 (72)
Female	23 (29)	24 (27)	47 (28)
Race			
Asian	39 (49)	44 (49)	83 (49)
Black	0	2 (2)	2 (1)
White	36 (45)	38 (43)	74 (44)
Combination of above/other	5 (6)	5 (6)	10 (6)
Country of residence			
Germany	17 (21)	15 (17)	32 (19)
Italy	0	5 (6)	5 (3)
Singapore	8 (10)	7 (8)	15 (9)
Thailand	27 (34)	31 (35)	58 (34)
United States	6 (8)	12 (14)	18 (11)
United Kingdom	22 (28)	19 (21)	41 (24)
CMA diagnosed by:			
Anaphylaxis history	9 (11)	9 (10)	18 (11)
DBPCFC	11 (14)	12 (14)	23 (14)
Open milk challenge	60 (75)	68 (76)	128 (75)
SPT wheal size for CM (stratification factor)			
0-5 mm	43 (54)	45 (51)	88 (52)
>=6 mm	37 (46)	44 (49)	81 (48)
CM-specific IgE level at baseline (kU/L), mean ± SD	28.7 ± 121.7	13.0 ± 34.2	20.4 ± 87.6
Mode of delivery			
Cesarean section	43 (54)	41 (46)	84 (50)
Vaginal	37 (46)	48 (54)	85 (50)
Family history of atopy			
At least 1 parent	64 (80)	64 (72)	128 (76)
Medical history of presenting allergy complaints of:			
Eczema	65 (81)	74 (83)	139 (82)
Acute urticaria	33 (41)	32 (36)	65 (39)
Wheezing	5 (6)	13 (15)	18 (11)
Dyspnea	3 (4)	7 (8)	10 (6)
Stridor	4 (5)	6 (7)	10 (6)
Dysphonia	1 (1)	2 (2)	3 (2)
Aphonia	1 (1)	1 (1)	2 (1)
Sneezing/congestion	17 (21)	19 (21)	36 (21)
Conjunctivitis	14 (18)	8 (9)	22 (13)
Severe abdominal symptoms	6 (8)	13 (15)	19 (11)
Change in behavior such as irritability	15 (19)	16 (18)	31 (18)
Sensitized to multiple foods (based on SPT results)	60 (75)	52 (58)	112 (66)
Subjects breast-fed at all			
Yes	79 (99)	85 (96)	164 (97)
No	1 (1)	4 (5)	5 (3)
Subjects exclusively breast-fed until study entry	6 (18)	8 (24)	14 (21)
Type of bottle feeding			
Whole protein (milk/soy)	37 (51)	48 (59)	85 (56)
Extensively hydrolyzed formula	39 (54)	47 (58)	86 (56)
AAF	51 (71)	59 (73)	110 (72)
Missing	8	8	16

Denominator to calculate percentage is number of subjects in treatment group with nonmissing data. Data are presented as no. (%) unless otherwise indicated

Supplementary Table 2. Median (Q1–Q3) (branched) short-chain fatty acids, intestinal inflammation and barrier markers and saliva markers at baseline, and 6, 12, 24 and 36 months after study initiation in children who received amino acid-based formula with synbiotics (AAF-S) and those who received amino acid-based formula without synbiotics (AAF) for 12 months.

	Baseline											6 months										
	AAF-S					AAF					AAF-S vs AAF	AAF-S					AAF					AAF-S vs AAF
	n	Med.	Q1	-	Q3	n	Med.	Q1	-	Q3	p-value	n	Med.	Q1	-	Q3	n	Med.	Q1	-	Q3	p-value
**Short-chain fatty acids**																						
Acetic acid (mmol/kg)	62	60.4	48.1	-	81.0	73	59.2	42.6	-	80.3	0.680	70	62.6	50.5	-	86.3	74	64.1	45.2	-	84.9	0.893
Butyric acid (mmol/kg)	62	5.0	2.2	-	7.7	73	6.5	2.9	-	10.3	0.101	70	8.2	4.2	-	14.8	74	8.7	6.5	-	15.0	0.193
Propionic acid (mmol/kg)	62	12.3	7.1	-	17.5	73	12.5	7.0	-	19.2	0.761	**70**	**11.5**	**6.8**	**-**	**16.8**	**74**	**14.3**	**10.8**	**-**	**21.3**	**0.004**
Valeric acid (mmol/kg)	62	0.0	0.0	-	0.5	73	0.0	0.0	-	0.3	0.409	**70**	**0.0**	**0.0**	**-**	**0.4**	**74**	**0.4**	**0.0**	**-**	**1.4**	**0.016**
Isobutyric acid (mmol/kg)	62	1.0	0.5	-	1.7	73	1.0	0.5	-	1.5	0.504	**70**	**0.8**	**0.0**	**-**	**1.4**	**74**	**1.3**	**0.7**	**-**	**2.1**	**0.010**
Isovaleric acid (mmol/kg)	62	1.5	0.6	-	2.4	73	1.3	0.6	-	1.9	0.366	**70**	**1.2**	**0.5**	**-**	**1.9**	**74**	**1.8**	**0.9**	**-**	**3.0**	**0.004**
Percentage Acetic acid of 6 SCFA (acet+prop+but+val+isobut+isoval) (%)	62	74.8	66.8	-	79.7	73	72.4	67.1	-	79.1	0.512	**70**	**73.2**	**66.1**	**-**	**79.1**	**74**	**65.9**	**61.2**	**-**	**72.6**	**<0.001**
Percentage Butyric acid of 6 SCFA (acet+prop+but+val+isobut+isoval) (%)	62	5.5	3.7	-	9.8	73	7.7	4.3	-	11.2	0.090	70	9.7	4.8	-	13.9	74	10.4	6.4	-	15.6	0.317
Percentage Propionic acid of 6 SCFA (acet+prop+but+val+isobut+isoval) (%)	62	15.7	10.4	-	20.2	73	14.9	9.0	-	19.0	0.974	**70**	**13.4**	**8.8**	**-**	**17.5**	**74**	**17.5**	**12.6**	**-**	**21.5**	**0.002**
Percentage Valeric acid of 6 SCFA (acet+prop+but+val+isobut+isoval) (%)	62	0.0	0.0	-	0.6	73	0.0	0.0	-	0.3	0.529	**70**	**0.0**	**0.0**	**-**	**0.4**	**74**	**0.4**	**0.0**	**-**	**1.7**	**0.045**
Percentage Isobutyric acid of 6 SCFA (acet+prop+but+val+isobut+isoval) (%)	62	1.3	0.5	-	2.2	73	1.2	0.5	-	1.8	0.498	**70**	**1.1**	**0.1**	**-**	**1.5**	**74**	**1.5**	**0.7**	**-**	**2.5**	**0.016**
Percentage Isovaleric acid of 6 SCFA (acet+prop+but+val+isobut+isoval) (%)	62	6.7	4.1	-	8.9	73	5.8	2.9	-	9.2	0.340	70	4.6	2.1	-	7.3	74	6.0	3.0	-	9.1	0.071
**Metabolites**																						
Lactic acid (mmol/kg)	62	0.2	0.2	-	4.9	73	0.2	0.2	-	2.2	0.360	**70**	**1.9**	**0.2**	**-**	**5.2**	**74**	**0.2**	**0.2**	**-**	**1.9**	**0.002**
**Intestinal inflammation and barrier markers**																						
Calprotectin (µg/g)	61	207.2	101.0	-	480.8	71	137.3	75.0	-	314.4	0.119	69	110.5	56	-	262.5	71	110.7	47.5	-	250.9	0.568
Secretory IgA (µg/g)	62	1042.5	303.2	-	2153.4	73	749.5	406.4	-	1515.8	0.398	70	857.4	40.3.1	-	1757.4	74	629.4	243.2	-	1451.5	0.218
Alpha-1 Antitrypsin (µg/mL)	65	982.4	619.4	-	1448.3	77	862.9	448.3	-	1367.4	0.427	71	626.3	358.2	-	999.2	76	731.3	442.4	-	1211.1	0.169
Eosinophil Derived Neurotoxin (ng/mL)	65	1823.0	875.0	-	4362.9	77	1698.4	964.7	-	2876.1	0.597	71	1062.0	584.8	-	2377.2	76	1270.6	821.7	-	2547.7	0.359
Eosinophil Cationic Protein (µg/L)	65	80.7	26.2	-	225.6	77	72.6	26.1	-	164.6	0.585	71	73.4	26.1	-	270.0	76	63.0	27.8	-	198.7	0.713
Fecal pH	63	6.5	5.7	-	6.9	73	6.4	5.9	-	6.8	0.981	**70**	**6.1**	**5.7**	**-**	**6.6**	**74**	**6.5**	**6.0**	**-**	**6.9**	**0.004**
**Saliva markers**																						
Secretory IgA (µg/mL)	72	72.4	41.1	-	153.7	79	73.2	36.8	-	112.0	0.586	64	122.4	57.5	-	172.9	68	92.7	49.6	-	152.1	0.247

Bold values represent significant differences between AAF-S and AAF groups.

Supplementary Table 2. continuation

	12 months											24 months										
	**AAF-S**					**AAF**					**AAF-S vs AAF**	**AAF-S**					**AAF**					**AAF-S vs AAF**
	**n**	**Med.**	**Q1**	**-**	**Q3**	**n**	**Med.**	**Q1**	**-**	**Q3**	**p-value**	**n**	**Med.**	**Q1**	**-**	**Q3**	**n**	**Med.**	**Q1**	**-**	**Q3**	**p-value**
**Short-chain fatty acids**																						
Acetic acid (mmol/kg)	63	58.6	48.8	-	75.2	72	63.4	49.1	-	77.0	0.416	62	50.3	35.3	-	65.8	59	51.5	37.9	-	68.0	0.606
Butyric acid (mmol/kg)	63	10.6	6.4	-	19.6	72	10.7	6.7	-	17.1	0.935	62	10.6	7.1	-	15.7	59	10.0	7.3	-	16.5	0.936
Propionic acid (mmol/kg)	63	12.6	9.6	-	20.4	72	15.0	9.8	-	21.9	0.377	62	13.3	9.3	-	18.1	59	14.6	10.6	-	20.6	0.216
Valeric acid (mmol/kg)	63	0.4	0.0	-	1.7	72	0.4	0.0	-	1.3	0.506	62	1.2	0.4	-	2.2	59	1.2	0.3	-	2.3	0.846
Isobutyric acid (mmol/kg)	63	1.0	0.5	-	2.2	72	1.2	0.6	-	1.9	0.332	62	1.6	0.9	-	2.3	59	1.5	0.7	-	2.1	0.416
Isovaleric acid (mmol/kg)	63	1.3	0.7	-	3.4	72	1.8	0.9	-	3.0	0.234	62	2.2	1.2	-	3.5	59	2.0	0.9	-	3.3	0.461
Percentage Acetic acid of 6 SCFA (acet+prop+but+val+isobut+isoval) (%)	63	66.5	60.8	-	71.2	72	65.2	60.0	-	71.7	0.729	62	59.6	55.1	-	67.1	59	61.0	54.8	-	67.1	0.915
Percentage Butyric acid of 6 SCFA (acet+prop+but+val+isobut+isoval) (%)	63	12.4	7.5	-	18.1	72	10.9	8.8	-	17.0	0.490	62	13.1	9.9	-	17.5	59	13.1	9.4	-	17.9	0.785
Percentage Propionic acid of 6 SCFA (acet+prop+but+val+isobut+isoval) (%)	63	15.4	10.6	-	19.5	72	17.1	12.0	-	19.4	0.303	62	19.5	13.2	-	21.4	59	18.2	13.7	-	22.0	0.891
Percentage Valeric acid of 6 SCFA (acet+prop+but+val+isobut+isoval) (%)	63	0.3	0.0	-	1.5	72	0.5	0.0	-	1.6	0.601	62	1.5	0.4	-	2.8	59	1.6	0.2	-	2.6	0.688
Percentage Isobutyric acid of 6 SCFA (acet+prop+but+val+isobut+isoval) (%)	63	1.1	0.5	-	2.0	72	1.4	0.8	-	2.1	0.251	62	1.9	1.3	-	3.1	59	1.8	0.9	-	2.9	0.349
Percentage Isovaleric acid of 6 SCFA (acet+prop+but+val+isobut+isoval) (%)	63	4.3	1.9	-	7.8	72	5.6	3.2	-	9.2	0.133	62	6.6	4.1	-	10.2	59	6.6	2.6	-	8.9	0.349
**Metabolites**																						
Lactic acid (mmol/kg)	63	2.5	1.9	-	3.2	72	2.5	1.9	-	3.1	0.662	62	0.2	0.2	-	0.2	59	0.2	0.2	-	0.2	0.978
**Intestinal inflammation and barrier markers**																						
Calprotectin (µg/g)	62	96.5	46.8	-	277.5	71	81.9	48.9	-	213.8	0.538	60	111.2	43.0	-	315.3	55	72.5	39.8	-	232.9	0.337
Secretory IgA (µg/g)	63	987.3	378.8	-	1509.7	72	970.8	402.2	-	2971.9	0.595	61	2588.3	1399.1	-	4906.9	59	3557.6	1579.5	-	5756.9	0.247
Alpha-1 Antitrypsin (µg/mL)	65	706.9	402.6	-	1012.3	76	606.9	401.2	-	1107.0	0.959	61	383	254.1	-	687.1	57	397.0	283.3	-	544.6	0.944
Eosinophil Derived Neurotoxin (ng/mL)	65	1118.8	432.0	-	1801.6	75	1025.2	557.2	-	1683.6	0.615	58	907.0	514.4	-	1589.6	58	883.6	382.0	-	1311.6	0.418
Eosinophil Cationic Protein (µg/L)	65	59.5	30.5	-	102.7	76	56.2	27.0	-	134.0	0.792	61	64.2	34.0	-	119.0	58	48.1	30.1	-	108.5	0.686
Fecal pH	64	6.2	5.8	-	6.7	73	6.2	5.9	-	7.0	0.310	62	6.3	5.8	-	6.9	59	6.4	6.0	-	6.8	0.903
**Saliva markers**																						
Secretory IgA (µg/mL)	66	120.1	78.8	-	225.7	77	111.8	68.3	-	179.0	0.137	53	108.0	57.0	-	156.0	64	99.0	79.0	-	133.5	0.755

Bold values represent significant differences between AAF-S and AAF groups.

Supplementary Table 2. continuation

	36 months										
	AAF-S					AAF					AAF-S vs AAF
	n	Med.	Q1	-	Q3	n	Med.	Q1	-	Q3	p-value
**Short-chain fatty acids**											
Acetic acid (mmol/kg)	54	45.6	29.7	-	63.1	60	53.0	39.6	-	64.6	0.083
Butyric acid (mmol/kg)	54	10.5	5.9	-	17.9	60	12.1	8.0	-	17.5	0.322
Propionic acid (mmol/kg)	54	13.4	10.0	-	19.8	60	16.7	10.4	-	20.7	0.178
Valeric acid (mmol/kg)	54	1.2	0.7	-	2.1	60	1.5	0.8	-	2.3	0.562
Isobutyric acid (mmol/kg)	54	1.7	1.0	-	2.2	60	1.7	1.0	-	2.2	0.989
Isovaleric acid (mmol/kg)	54	2.4	1.4	-	3.5	60	2.5	1.3	-	3.6	0.851
Percentage Acetic acid of 6 SCFA (acet+prop+but+val+isobut+isoval) (%)	54	57.6	52.4	-	63.4	60	60.2	55.2	-	65.7	0.150
Percentage Butyric acid of 6 SCFA (acet+prop+but+val+isobut+isoval) (%)	54	14.0	10.8	-	20.1	60	14.2	10.8	-	18.3	0.787
Percentage Propionic acid of 6 SCFA (acet+prop+but+val+isobut+isoval) (%)	54	19.4	13.8	-	23.5	60	19.2	14.1	-	22.8	0.761
Percentage Valeric acid of 6 SCFA (acet+prop+but+val+isobut+isoval) (%)	54	2.0	0.8	-	3.0	60	1.7	0.9	-	2.8	0.490
Percentage Isobutyric acid of 6 SCFA (acet+prop+but+val+isobut+isoval) (%)	54	2.5	1.3	-	3.7	60	1.9	1.3	-	2.7	0.122
Percentage Isovaleric acid of 6 SCFA (acet+prop+but+val+isobut+isoval) (%)	54	7.8	4.3	-	11.7	60	6.7	4.3	-	9.6	0.201
**Metabolites**											
Lactic acid (mmol/kg)	54	1.0	0.2	-	4.5	60	2.0	0.2	-	5.8	0.439
**Intestinal inflammation and barrier markers**											
Calprotectin (µg/g)	54	79.5	35.4	-	162.2	62	107.5	59.5	-	235.3	0.154
Secretory IgA (µg/g)	54	1142.1	292.2	-	3237.6	60	1022.1	295.3	-	2821.6	0.660
Alpha-1 Antitrypsin (µg/mL)	52	607.1	328.2	-	765.1	59	499.2	315.4	-	803.3	0.899
Eosinophil Derived Neurotoxin (ng/mL)	51	768.0	483.0	-	1319.0	57	911.0	509.0	-	1254.0	0.477
Eosinophil Cationic Protein (µg/L)	51	47.0	26.0	-	157.0	59	65.5	40.0	-	175.0	0.181
Fecal pH	55	6.5	6.1	-	6.9	62	6.7	6.2	-	6.9	0.714
**Saliva markers**											
Secretory IgA (µg/mL)	64	90.5	43.5	-	174.5	66	71.0	33.0	-	166.0	0.225

Bold values represent significant differences between AAF-S and AAF groups.
